# Prevalence and correlates of post-stroke anxiety in Changde, China during 2023 following the lifting of COVID-19 restrictions

**DOI:** 10.3389/fpsyt.2024.1430034

**Published:** 2024-09-30

**Authors:** Shangyu Luo, Yunjun Hong, Jun Wen, Xiaobo Zhang

**Affiliations:** Changde Hospital, Xiangya School of Medicine, Central South University (The First People’s Hospital of Changde City), Changde, Hunan, China

**Keywords:** COVID-19, stroke, anxiety, hyperlipidemia, migraine

## Abstract

**Background:**

Studies on post-stroke anxiety (PSA) following the lifting of COVID-19 restriction measures are currently lacking. We investigated the factors affecting PSA after full release of COVID-19 epidemic in China.

**Methods:**

Patients with stroke admitted to the First People’s Hospital of Changde City from March 2023 to September 2023 participated in a questionnaire survey comprising a general demographic questionnaire, the Generalized Anxiety Scale-7. Additionally, data on the National Institutes of Health Stroke Scale, modified Rankin Scale, C-reactive protein (CRP), thyroid-stimulating hormone (TSH), homocysteine, TOAST classification, and the stroke site were collected, and the correlations between these indices and the mental health conditions of the patients were evaluated.

**Results:**

Among 947 patients, the incidence of PSA was 14.57%.PSA was not linked to prior COVID-19 infection. This study found that Sleep duration (P=0.01), hyperlipidemia (P=0.01), migraine (P=0.02), and family history of stroke (P=0.01) were associated with PSA.

**Conclusions:**

Our study found that the prevalence of PSA was 14.57%. In addition, sleep duration, hyperlipidemia, migraine and family history of stroke were independent risk factors for PSA following the lifting of COVID-19 restrictions.

## 1 Introduction

Novel coronavirus pneumonia, also known as coronavirus disease 2019 (COVID-19), is a widespread infectious disease posing a serious global threat to human health. As of March 2022, more than 445 million infections and 6 million fatalities were reported worldwide (1). After the initial outbreak in December 2019, China has diligently adhered to strict defense measures and a dynamic zero-COVID policy. Considering the gradual decrease in pathogenicity in Considering the decrease in pathogenicity towards the end of 2022,The Joint Prevention and Control Mechanism Comprehensive Team of The State Council issued the “New Ten Measures” on December 7, 2022 (2). Subsequently, the number of positive cases decreased after peaking on December 22,2022 (6.94 million cases) (3).

Stroke is the second highest cause of mortality globally and among the primary contributors to disability (4). Post-stroke anxiety (PSA) is a frequent and debilitating consequence experienced by a significant proportion of stroke survivors. It is the result of motor dysfunction and severe decline in quality of life, which can lead to a continuous and stable decline in quality of life. However, compared with other post-stroke psychological disorders, PSA is currently relatively neglected as a serious psychological and physical problem (5). PSA frequently presents as an exaggerated emphasis on individual outcomes, encompassing stroke recurrence, resumption of occupational activities, falls prevention, and the preservation of autonomy (6). Studies have demonstrated that COVID-19 infection or suspected infection can trigger intense emotional and behavioral responses, including fear, boredom, loneliness, anxiety, insomnia, or anger (7). The prevalence and burden of depression and anxiety have increased significantly during the COVID-19 pandemic (8). However, studies conducted during the COVID-19 pandemic period have indicated a comparable prevalence of post-stroke anxiety and depression to that observed in non-pandemic periods (9).

However, there are currently no studies on PSA after the full release of COVID-19. Therefore, further studies are needed to investigate whether COVID-19 infection is associated with PSA, and the related risk factors of PSA after full release of COVID-19 epidemic.

In order to clarify the above issues, the cross-sectional study investigated the factors associated with the development and persistence of anxiety following stroke after COVID-19 opening, which will provide a basis for early screening and intervention of PSA.

## 2 Methods

### 2.1 Study design and patient selection

**Figure 1:**
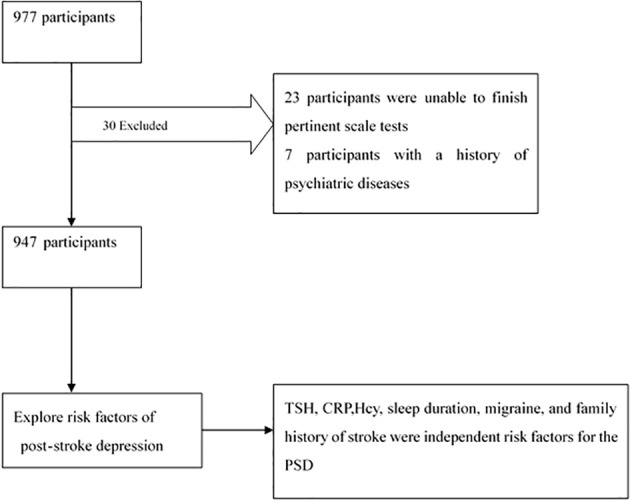
Flow chart of study.

This study adopted a cross-sectional research design. We calculated the sample size by sample size = (Z value * standard deviation/error) ^2.All patients with stroke (including cerebral hemorrhage and infarction) admitted to the neurology department of the First People’s Hospital of Changde City from March 2023 to September 2023 were included in this study (**Figure 1**).

The patient inclusion criteria were as follows:

1. cerebral infarction or hemorrhage diagnosed according to the International Statistical Classification of Diseases and Related Health Problems, 10th Revision (ICD-10) and
2. site of hemorrhage or infarction identified by computed tomography (CT) or magnetic resonance imaging (MRI).

The patient exclusion criteria were as follows:

1. medical history of a psychiatric disorder,
2. unable to complete the questionnaire survey, or
3. opted out of the study.

Each patient underwent the below evaluations.

1. Laboratory examination: Standard blood tests, including the measurement of serum thyroid-stimulating hormone (TSH), homocysteine (Hcy), and C-reactive protein (CRP) levels, were conducted during admission.
2. Stroke-related assessments: The assessments included the patients’ scores on the National Institutes of Health Stroke Scale (NIHSS) and modified Rankin Scale (mRS), estimation of the bleeding volume and stroke location, and the Trial of ORG 10172 in Acute Stroke Treatment (TOAST) subtype staging using various imaging and clinical data.
3. Web-based questionnaire survey: We administered face-to-face questionnaires to the patients. The web-based questionnaire survey on the Questionnaire Star platform collected information such as sex, age, height, weight, employment status, marital status, education level, presence of post-stroke pain, smoking and alcohol habits, neocoronary infections, past medical history, and family history of psychiatric disorders and stroke, as well as included assessments using the Generalized Anxiety Scale-7 (GAD-7).

#### 2.1.1 Standard blood tests

##### 2.1.1.1 TSH estimation

Fasting blood samples were collected at 6 a.m. on the day after admission. Samples were collected into standardized tubes containing an anticoagulant (EDTA), and stored at −80°C. The TSH levels were measured using a standardized radioimmunoassay kit. The normal range of TSH was 0.35–4.94 μIU/mL.

##### 2.1.1.2 Hcy estimation

Serum Hcy levels were measured via an enzyme cycling method with a normal range of 0–15 μmol/L.

##### 2.1.1.3 CRP estimation

Serum CRP levels were quantitatively determined via immunoturbidimetry within a detection range of 0–10 mg/L.

#### 2.1.2 Stroke-related assessments

##### 2.1.2.1 NIHSS

The NIHSS score is used to assess stroke severity (10). The total NIHSS score ranges from 0 to 42, wherein 0 indicates normal function and higher scores imply increasing impairment.

##### 2.1.2.2 mRS

The mRS is used to examine residual disability in patients with stroke. The mRS is scored on a 5-point scale, with 0 representing no symptoms and 5 suggesting severe disability. The validity as well as interobserver and intraobserver reliability of the mRS in scoring the residual disability of patients with stroke in terms of the amount of assistance required to achieve various degrees of autonomy have been well documented (10).

##### 2.1.2.3 TOAST classification system

The TOAST classification system includes five categories: 1) large-artery atherosclerosis, 2) cardioembolism, 3) small-artery occlusion (lacune), 4) stroke of other determined etiology, and 5) stroke of undetermined etiology (**Table 1**). Diagnoses are based on clinical features and data collected via tests such as brain imaging (CT/MRI), cardiac imaging (including echocardiography), duplex sonography of the extracranial arteries, arteriography, and laboratory assessments for a prothrombotic state (11).

**Table 1:** Demographic characteristics of stroke patients.

Variables	Category	N (%)/median (quartile)
Gender	Male	608 (64.2%)
	Female	339 (35.8%)
Age		64.5 (15)
Marital Status	be married	941 (99.40)
	dissociaton	3 (0.30)
	singlehood	2 (0.20)
	widowhood	1 (0.10)
	employment	140 (14.8%)
employment status	retirement	300 (31.7%)
	unemployed	507 (53.5%)
	Junior high school and below	763 (80.7%)
Educational Status	College or university	58 (6.1%)
	High school or technical secondary school	125 (13.2%)
	Junior high school and below	763 (80.7%)

#### 2.1.3 Questionnaire

##### 2.1.2.1 GAD-7

The Chinese version of the GAD-7 scale was employed to evaluate the anxiety levels of the patients. The GAD-7 is a 7-item scale that determines the severity of anxiety symptoms on a scale of 0 (none at all) to 3 (almost every day). The total GAD-7 score ranges from 0–21, with higher scores implying severe anxiety. In line with prior studies, a total score of ≥5 was suggestive of a state of anxiety. Finally, the degree of anxiety based on the total score was categorized as follows: mild (5–9 points), moderate (10–14 points), and severe anxiety (≥15 points) (12–14).

### 2.2 Statistical analysis

All data analyses were performed using IBM SPSS software package version 20.0. Continuous numerical variables with normal distribution were presented as mean ± standard deviation, whereas quantitative and rank data with non-normal distribution were expressed as median (interquartile spacing). An independent sample t-test was utilized to compare normally distributed data, while the rank sum test was employed to compare non-normally distributed quantitative data. Count data were reported as quantity (%). The chi-square test was used to compare differences, and binary logistic regression was applied to explore the independent risk factors for anxiety. A P-value of <0.05 was considered statistically significant.

## 3 Results

### 3.1 Sociodemographic and clinical characteristics

A total of 947 participants, including men and women, were enrolled for six months during the study. The participants’ demographic, clinical, GAD-7 scores were in **Tables 1**, **2**. Our study found that the prevalence of anxiety was 14.57%, the mean GAD-7 scores was 1.38 ± 2.89. 138. Among them, 112 cases (11.83%) had mild anxiety, 20 cases (2.11%) had moderate anxiety, and 6 cases (0.63%) had severe anxiety.

**Table 2:** Clinical characteristics of stroke patients.

Variables	Category	N(%)/median (quartile)/ Mean ± SD
Type of stroke	Ischemic stroke	860 (90.8%)
	Hemorrhagic stroke	87 (9.2%)
	brainstem	158 (16.7%)
	Bilateral cerebral hemispheres	105 (11.1%)
Lesion site	Parencephalon	40 (4.2%)
	Right cerebral hemisphere	320 (33.8%)
	Left cerebral hemisphere	323 (34.1%)
	Large vessel atherosclerosis	590 (68.8%)
The amount of bleeding		4 (5.75)
NIHSS score		2 (8.75)
MRS score		4 (2)
TOAST Classification	Small vessel occlusion	203 (23.7%)
	Cardio-embolic source	45 (5.2%)
	Undetermined etiologies	20 (2.3%)
	>6 months	14 (1.5%)
Time since stroke	>7days ≤ 6 months	535 (56.5%)
	≤7days	398 (42%)
feel pain after a stroke	yes	79 (8.3%)
	no	868 (91.7%)
	<5 hours	177 (18.7%)
pre-stroke sleep duration	5~6hours	135 (14.3%)
	6~7 hours	72 (7.6%)
	>7 hours	563 (59.5%)
	No	596 (62.9%)
BMI		24.43 ± 5.11
Height		163 (11)
Weight		65 (16)
Homocysteine		14.405 (6.71)
TSH		2.0925 (2.22)
CRP		3.25 (7.33)
Infected with COVID-19	Currently infecting	0 (0%)
	No	287 (30.5%)
History of Smoking	Current smoker	307 (32.4%)
	Previous smoker	44 (4.6%)
	No	805 (85%)
History of alcohol drinking	Currently drinking	115 (12.1%)
	Previously drinking	27 (2.9%)
	Previously infecting	654 (69.5%)
History of hypertension		686 (72.4%)
History of hyperlipemia		113 (11.9%)
History of diabetes		283 (29.9%)
History of heart disease		191 (20.2%)
History of stroke		219 (23.1%)
History of chronic nephrosis		134 (14.1%)
History of hemicrania		16 (1.7%)
Family history of mental disorders		2 (0.2%)
Family history of stroke		29 (3.1%)
GAD-7		1.38 ± 2.89. 138

NIHSS, Neurological Institute of Health Stroke Scale; mRS, Modified Rankin Scale; pre-stroke sleep duration, Sleep duration 1 month before stroke; BMI, Body Mass Index; TSH, Thyroid-stimulating hormone; CRP, C-reactive protein; GAD-7, Generalized Anxiety Scale-7.

### 3.2 Factors related to patient anxiety

In order to explore the related factors of anxiety in stroke patients, patients were divided into groups according to the score of GAD-7, GAD-7 < 5 points were no anxiety and GAD-7 ≥ 5 points were anxiety group. The different parameters of anxiety group in no anxiety group are shown in **Tables 3**, **4**. As shown in **Tables 3**, **4**, compared with the non-anxiety group, the anxiety group was younger (59.92 ± 10.72, P=0.01), taller (162.85 ± 7.18, P=0.01), higher BMI (21.80(6.6), P=0.01), and higher proportion of cerebral infarction (P=0.01). More patients with stroke duration ≤7 days (P=0.01), more patients with employment (P=0.01), more patients with post-stroke pain (P=0.01), more patients with sleep duration < 5 hours (P=0.01), and more patients with sleep duration > 7 hours (P=0.01) than those without anxiety. The proportion of previous alcohol use was higher in the anxiety group (P=0.02), whereas the proportion of non-alcohol use and current alcohol use was higher in the non-anxiety group. The proportion of family history of hyperlipidemia, migraine and stroke was higher in the anxiety group (P=0.01).

**Table 3:** Association between post-stroke anxiety and demographic data.

Category	No anxiety	Anxiety	T/χ2	P
Gender			0.09	0.76
Male	521 (64.4)	87 (63)		
Female	288 (35.6)	51 (37)		
Age (years)	66.79 ± 10.69	59.92 ± 10.72	6.97	0.01
employment status			21.99	0.01
employment	102 (12.6)	38 (27.5)		
retirement	268 (33.1)	32 (23.2)		
unemployed	439 (54.3)	68 (49.3)		
Educational Status			1.80	0.41
Junior high school and below	658 (81.3)	105 (76.6)		
College or university	47 (5.8)	11 (8)		
High school or technical secondary school	104 (12.9)	21 (15.3)		

**Table 4:** Association between post-stroke anxiety and clinical characteristics of stroke patients.

Category	No anxiety	Anxiety	T/Z/χ2	P
Type of stroke			5.99	0.01
Ischemic stroke	727 (89.9)	133 (96.4)		
Hemorrhagic stroke	82 (10.1)	5 (3.6)		
Lesion site			1.07	0.90
Bilateral cerebral hemispheres	136 (16.8)	22 (15.9)		
Parencephalon	89 (11)	16 (11.6)		
Right cerebral hemisphere	36 (4.5)	4 (2.9)		
Left cerebral hemisphere	270 (33.4)	50 (36.2)		
Bilateral cerebral hemispheres	277 (34.3)	46 (33.3)		
The amount of bleeding (ml)	5 (8)	5 (6)	-0.17	0.87
NIHSS score	2 (3)	2 (3)	0.38	0.7
mRS score	2 (2)	2 (1)	0.57	0.57
TOAST Classification			6.20	0.10
Larg evessel therosclerosis	493 (68)	97 (72.9)		
Small vessel occlusion	174 (24)	29 (21.8)		
Cardio-embolic source	43 (5.9)	2 (1.5)		
Undetermined etiologies	15 (2.1)	5 (3.8)		
Time since stroke			9.57	0.01
>6 months	9 (1.1)	5 (3.6)		
>7days ≤ 6 months	470 (58.1)	65 (47.1)		
≤7days	330 (40.8)	68 (49.3)		
feel pain after a stroke			23.28	0.01
yes	53 (6.6)	26 (18.8)		
no	756 (93.4)	112 (81.2)		
pre-stroke sleep duration			19.64	0.01
<5 hours	137 (16.9)	40 (29)		
5~6hours	116 (14.3)	19 (13.8)		
6~7 hours	55 (6.8)	17 (12.3)		
>7 hours	501 (61.9)	62 (44.9)		
BMI	24.61 (6.4)	21.80 (6.6)	-3.27	0.01
Weight	64.5 (16)	62.5 (15)	-0.19	0.85
Height	162.74 ± 7.40	162.85 ± 7.18	-0.12	0.01
TSH	2.03 (1.99)	1.83 (1.89)	-0.95	0.34
CRP	3 (6.9)	3 (13.02)	1.84	0.07
Homocysteine	13.91 (6.74)	14.17 (7.67)	0.88	0.38
Infected with COVID-19			0.01	0.99
Previously infecting	558 (69.5)	96 (69.6)		
No	245 (30.5)	42 (30.4)		
History of Smoking			2.33	0.31
No	516 (63.8)	80 (58)		
Current smoker	258 (31.9)	49 (35.5)		
Previous smoker	35 (4.3)	9 (6.5)		
History of alcohol drinking			7.96	0.02
No	691 (85.4)	114 (82.6)		
Currently drinking	100 (12.4)	15 (10.9)		
Previously drinking	18 (2.2)	9 (6.5)		
History of hypertension			0.39	0.53
No	226 (27.9)	35 (25.4)		
Yes	583 (72.1)	103 (74.6)		
History of hyperlipemia			10.77	0.01
No	724 (89.5)	110 (79.7)		
Yes	85 (10.5)	28 (20.3)		
History of diabetes			0.31	0.58
No	570 (70.5)	94 (68.1)		
Yes	239 (29.5)	44 (31.9)		
History of heart disease			0.18	0.67
No	644 (79.6)	112 (81.2)		
Yes	165 (20.4)	26 (18.8)		
History of stroke			0.21	0.65
No	624 (77.1)	104 (75.4)		
Yes	185 (22.9)	34 (24.6)		
History of chronic nephrosis			0.15	0.70
No	696 (86)	117 (84.8)		
Yes	113 (14)	21 (15.2)		
History of hemicrania			11.13	0.01
No	800 (98.9)	131 (94.9)		
Yes	9 (1.1)	7 (5.1)		
Family history of mental disorders			2.02	0.15
No	808 (99.9)	137 (99.3)		
Yes	1 (0.1)	1 (0.7)		
Family history of stroke			27.30	0.01
No	794 (98.1)	124 (89.9)		
Yes	15 (1.9)	14 (10.1)		

NIHSS, Neurological Institute of Health Stroke Scale; mRS, Modified Rankin Scale; pre-stroke sleep duration, Sleep duration 1 month before stroke; BMI, Body Mass Index; TSH, Thyroid-stimulating hormone; CRP, C-reactive protein.

Further univariate and multivariate logistic regression analysis showed that Sleep duration before stroke, hyperlipidemia, migraine, family history of stroke, and anxiety were independent risk factors for the occurrence of stroke (**Table 5**).

**Table 5:** Single-factor and multiple-factor logistic regression results.

Anxiety	Single factor regression		Multivariate regression	
	*OR (95 CI%)*	*P*	*OR (95 CI%)*	*P*
Age	0.94 (0.93,0.96)	0.01	0.97 (0.94,1)	0.74
Height	1 (0.97,1.04)	0.9	1 (0.96,1.04)	1.00
BMI	0.93(0.87,0.98)	0.01	0.98 (0.86,1.11)	0.99
Type of stroke	0.33 (0.13,0.84)	0.02	0.53 (0.18,1.59)	0.26
Time since stroke	1.25 (0.88,1.76)	0.21	0.83 (0.49,1.4)	0.48
employment status				
employment	1	0.01	1	0.37
retirement	0.32 (0.19,0.54)		0.59 (0.23,1.47)	
unemployed	0.42 (0.27,0.65)		0.57 (0.26,1.25)	
feel pain after a stroke	0.3 (0.18,0.5)	0.01	0.52 (0.24,1.12)	0.10
pre-stroke sleep duration	0.77 (0.67,0.89)	0.01	0.75 (0.6,0.93)	0.01^*^
History of alcohol drinking	1.36 (0.95,1.95)	0.09	1.47 (0.91,2.37)	0.12
History of hyperlipemia	2.17 (1.35,3.48)	0.01	2.32 (1.22,4.42)	0.01^*^
History of hemicrania	4.75 (1.74,12.97)	0.01	5.44 (1.32,22.52)	0.02^*^
Family history of stroke	5.98 (2.82,12.68)	0.01	3.65 (1.51,8.8)	0.01^*^

pre-stroke sleep duration, Sleep duration 1 month before stroke; BMI, Body Mass Index; ^*^P < 0.05.

## 4 Discussion

A total of 947 patients were enrolled in this cross-sectional study to explore the influencing factors of PSA after full release of COVID-19 epidemic. The present study revealed the prevalence of post-stroke anxiety was 14.57%. Sleep duration, hyperlipidemia, migraine and family history of stroke were independent risk factors for PSA.

Several studies have shown that during the COVID-19 pandemic, the prevalence rate of PSA is as high as 30.1% -32% (15, 16), which is significantly higher than our research results. The reason for the significant difference in results may be due to differences in research subjects, stroke onset time, and diagnostic tools used. The more important reason is that our investigation was conducted in the post pandemic era. The public was not so panicked about COVID-19 at this time.

This study suggests that pre-stroke sleep duration is associated with PSA, which is consistent with previous findings (17, 18), Improving sleep had a significant effect on anxiety (19). In sufficient sleep duration can impede the functioning of the prefrontal cortex, anterior cingulate cortex, amygdala, and striatum, thereby impacting emotion regulation. Additionally, it can disrupt the brain’s reward system through elevated levels of tumor necrosis factor-α (TNF-α), interleukin-6 (IL-6), and CRP, increased cortisol levels, hypothalamic-pituitary-adrenal axis imbalance, and exacerbation of inflammatory response. All these alterations are secondary to sleep insufficiency and contribute to heightened anxiety levels (20). Another mechanism involves sleep deprivation-induced reduction in the availability of dopamine D2/D3 receptors in the striatum, as dopamine, a monoamine neurotransmitter, plays a pivotal role in modulating the reward system and facilitating pleasurable experiences (21).Therefore, we need to pay attention to the sleep condition of stroke patients and intervene in time to reduce the occurrence of PSA.

Next, the current investigation revealed a significant association between hyperlipidemia and post-stroke anxiety, which is consistent with the findings of prior research studies (22, 23). Some studies have identified a positive correlation between anxiety and elevated triglyceride levels (24). Another study demonstrated the effectiveness of interventions targeting triglyceride levels in alleviating symptoms of anxiety (25). A recently published experimental animal study also revealed that an 8-week high-fat diet effectively induced metabolic disorders, including obesity and hyperlipidemia, leading to anxiety-like behavior in mice (26). The aforementioned studies suggest that dyslipidemia may contribute to anxiety, possibly due to the promotion of brain-derived neurotrophic factor (BDNF) production in salivary glands by hypertriglyceridemia, thereby increasing plasma BDNF content. Salivary BDNF influences the hippocampus and enhances anxiety-like behavior (27). Although a Mendelian randomization analysis conducted in 2023 found no causal relationship between lipids and anxiety (28), lipids are likely predictors of post-stroke depression PSA, and lipid-lowering therapy may improve PSA outcomes.

This study also observed an association between migraine history and PSA, which is consistent with the results of previous studies (29). Due to the impact on daily life, work, and interpersonal relationships, individuals with migraines often experience negative emotions such as anxiety and depression (30).In addition, depression and migraine share many pathological mechanisms, including: central nervous system morphology and dysfunction, neurotransmitter and receptor systems, hormonal regulation, neuroinflammation, environmental factors, genetic predisposition, personality and temperament (31).Therefore, we need to pay attention to stroke patients with a history of migraine, who may be more likely to develop PSD.

Lastly, our study has revealed that a family history of stroke is associated with anxiety, which may be attributed to the high incidence, mortality and recurrence rate of stroke. These factors impose a significant burden on caregivers of stroke patients, leading to increased rates of anxiety and depression among them (32), Consequently, individuals with a family history of stroke are more likely to face greater economic and life pressures, thereby increasing their susceptibility to anxiety.

### 4.1 Limitations

This study has a few limitations that should be considered. First, establishing a causal relationship between pertinent factors and PSA was challenging, owing to the cross-sectional nature of our study. Second, the long-term influence of COVID-19 infection on PSA must be evaluated using a longitudinal cohort investigation. Third, the incidence of cerebral hemorrhage was limited in our study sample. Finally, this study only utilized scale scores as the diagnostic criteria for PSA, potentially leading to some bias. Therefore, further investigation is warranted to examine the correlation between PSA and COVID-19, identify predictors for PSD, and explore the related pathogenic factors.

## 5 Conclusions

Our study found that the prevalence of PSA was 14.57% and sleep duration, hyperlipidemia, migraine and family history of stroke were independent risk factors for PSA following the lifting of COVID-19 restrictions. These conclusions have guiding significance for early detection and diagnosis of post-stroke anxiety in our clinic. Further studies are needed to determine the causal relationship between these risk factors and PSA, so that some preventive measures can be taken against PSA.

## Data Availability

The raw data supporting the conclusions of this article will be made available by the authors, without undue reservation.
